# Benefits of the Mediterranean Diet–Wine Association: The Role of Ingredients

**DOI:** 10.3390/molecules27041273

**Published:** 2022-02-14

**Authors:** Paula Silva, Norbert Latruffe

**Affiliations:** 1 Laboratory of Histology and Embryology, Institute of Biomedical Sciences Abel Salazar (ICBAS), Rua de Jorge Viterbo Ferreira nº228, 4050-313 Porto, Portugal; 2 iNOVA Media Lab, ICNOVA-NOVA Institute of Communication, NOVA School of Social Sciences and Humanities, Universidade NOVA de Lisboa, 1069-061 Lisbon, Portugal; 3 BioPeroxIL, INSERM, Biochemistry of Peroxisome and Inflammation & Lipid Metabolism, EA7270, University Bourgogne-Franche Comte, F-21000 Dijon, France

The cultural and nutritional aspects of the multi-secular Mediterranean civilization include diet as a central element of health and well-being, including wine if it is used in moderation. Indeed, Mediterranean meals provide food microcomponents including polyphenols, vitamins, fibers, polyunsaturated fatty acids, and oligoelements present in fruits, vegetables, olive oil, fish, infusions, etc. In addition, wine, especially the red variety, provide additional unique polyphenols with antioxidant properties, such as resveratrol, procyanidins, and monophenols, including hydroxytyrosol and tyrosol [1].

The Mediterranean diet is a model of eating based on the traditional foods and drinks of the countries surrounding the Mediterranean Sea. In recent decades, it has been promoted worldwide (UNESCO 2010) as one of the healthiest dietary patterns and has been reported to have benefits regarding chronic diseases, i.e., cardiovascular illness [2,3], breast [4] and colon cancer [5], cognition [6] and longevity [7]. Many consumers do not know what to believe when it comes to the health effects of drinking wine. Some researchers have associated moderate drinking with various health benefits, especially since wine is rich in antioxidants. Others, on the other hand, claim that even an occasional drink is harmful to health.

The publication of a Special Issue in the journal *Molecules* that focuses on the role of wine ingredients in the health benefits associated with the Mediterranean diet was a good opportunity to clarify this issue. Researchers were invited to submit manuscripts related to polyphenols, resveratrol, ageing, antioxidant, wine, health, the Mediterranean diet, nutrition, diseases, welfare, behavior, etc. at the level of mechanisms, analysis, and experimental and epidemiological studies.

The objective of this Special Issue is to highlight wine as part of the Mediterranean diet, especially through the perspectives of policymakers, the medical world, and the vectors of image.

The valuable accepted and published papers contributing to this Special Issue are as follows:

Original papers include ‘Resveratrol Hinders Postovulatory Aging by Modulating Oxidative Stress in Porcine Oocytes’ by Benazir Abbasi et al.; ‘Red Wine Extract Inhibits VEGF Secretion and Its Signaling Pathway in Retinal ARPE-19 Cells to Potentially Disrupt AMD’ by Clarisse Cornebise et al.; ‘Synthesis and Characterization of Novel Resveratrol Butyrate Esters That Have the Ability to Prevent Fat Accumulation in a Liver Cell Culture Model’ by You Lin Tain; ‘Protective Effects of Some Grapevine Polyphenols against Naturally Occurring Neuronal Death’ by Laura Lossi et al.; ‘Prevention of 7-Ketocholesterol-Induced Overproduction of Reactive Oxygen Species, Mitochondrial Dysfunction and Cell Death with Major Nutrients (Polyphenols, ω3 and ω9 Unsaturated Fatty Acids) of the Mediterranean Diet on N2a Neuronal Cells’ by Aline Yammine et al.

Additionally, several review papers are included—namely, ‘The Neuroprotective Role of Polydatin: Neuropharmacological Mechanisms, Molecular Targets, Therapeutic Potentials, and Clinical Perspective’ by Sajad Fakhri et al.; ‘Effects of Wine Components in Inflammatory Bowel Diseases’ by Josip Vrdoljak et al.; ‘Wine, Polyphenols, and Mediterranean Diets. What Else Is There to Say?’ by Celestino Santos-Buelga et al.; ‘Wine Intake in the Framework of a Mediterranean Diet and Chronic Non-Communicable Diseases: A Short Literature Review of the Last 5 Years’ by Simona Minzer et al.; ‘Wine’s Phenolic Compounds and Health: A Pythagorean View’ by Francesco Visioli et al.; ‘Wine Consumption and Oral Cavity Cancer: Friend or Foe, Two Faces of Janus’ by Paula Silva et al.

Many readers would be interested in the content amassed in this Special Issue, including scientists in nutrition, medical doctors, wine biochemists, wine tasting associations, winemakers, researchers in Vin viticulture and in enology, etc.

Notably, the topic of this Special Issue induced Science & Wine to promote the Second World Congress “Wine and Olive Oil Production: The Fluid Aspect of Mediterranean Diet” held on 2 to 3 June 2021, https://www.science-and-wine-conferences.com.
